# Supplementary material for: Determinants of Continued Breastfeeding at 12 and 24 Months: Results of an Australian Cohort Study
Source: Int J Environ Res Public Health. 2019 Oct 18;16(20):3980. doi: 10.3390/ijerph16203980 (PMC6843256; doi:10.3390/ijerph16203980)
Supplement: Supplementary file 1 [file ijerph-16-03980-s001.pdf]

Table S1. Comparison of characteristics of participants and non-participants and South Australian (SA) pregnancy outcome data for 2013.

	**Participant (n = 1450)**			**Non-Participants (n = 662)**			**SA Pregnancy Outcome 2013 (n = 19,925)**		
	%	95% CI		%	95% CI		%	95% CI	
Maternal age (yrs)									
<25	13.1	11.4	14.8	22.1	18.9	25.3	17.8	17.2	18.4
25–29	32.6	30.2	35.0	29.2	25.7	32.7	29.4	28.7	30.1
30+	54.1	51.5	56.7	47.7	43.9	51.5	52.7	51.7	53.7
Maternal education									
High school/vocational	48.8	46.2	51.4	64.8	61.2	68.4	N/A		
Some [a] university or graduate	50.8	48.2	53.4	34.9	31.3	38.5			
IRSAD[b] deciles									
IRSAD deciles 1–2	19.1	17.1	21.1	27.9	24.5	31.3	N/A		
IRSAD deciles 3–4	20.8	18.7	22.9	21.9	18.7	25.1			
IRSAD deciles 5–6	19.7	17.7	21.7	15.9	13.1	18.7			
IRSAD deciles 7–8	18.7	16.7	20.7	17.5	14.6	20.4			
IRSAD deciles 9–10	21.3	19.2	23.4	13.7	11.1	16.2			
Mother's country of birth									
Australia/New Zealand	71.4	69.1	73.7	63.1	59.4	66.8	76.5	75.4	77.6
UK	3.6	2.6	4.6	3.8	2.3	5.3	2.5	2.3	2.7
China	7.6	6.2	9.0	2.3	1.2	3.4	2.2	2.0	2.4
India	3.4	2.5	4.3	11.5	9.1	13.9	4.0	3.7	4.3
Rest of Asia	7.2	5.9	7.3	10.7	8.3	13.1	6.4	6.1	6.7
Other	6.1	4.9	7.3	7.9	5.8	10.0	5.8	5.5	6.1

[a] Commenced but did not complete University. [b] IRSAD: Index of Relative Socio-Economic Advantage and Disadvantage with decile 1 = most disadvantaged and 10 = most advantaged BF.

Table S2 Sensitivity analysis: Factors independently associated with breastfeeding at 12 months and 24 months postpartum with age of return to work removed

Characteristics	BF at 12 months [a] (n = 1141)		BF at 24months [a] (n = 1281)	
	**AOR**	**95%CI**	AOR	95%CI
Maternal characteristics				
Age (years)				
<25	0.49	0.24–0.99	0.50	0.15–1.60
25–34	0.84	0.57–1.24	0.64	0.37–1.13
≥35	1.00		1.00	
Highest level of education				
High school/vocational	1.00		1.00	
Some [a] university or graduate	2.23	1.57–3.16	1.17	0.66–2.08
Country of birth				
Australian & New Zealand	1.00		1.00	
UK/Ireland	0.53	0.21–1.32	1.78	0.61–5.20
India	0.84	0.49–1.46	1.96	0.88–4.36
China	1.55	0.75–3.21	1.57	0.43–5.75
Other Asia	1.06	0.59–1.89	3.31	1.58–6.93
Other	1.46	0.77–2.75	1.79	0.78–4.13
Pre-pregnancy BMI (kg/m^2)				
<25	1.00		1.00	
≥25	0.57	0.41–0.79	0.66	0.38–1.15
Parity				
Primiparous	1.00		1.00	
Multiparous	1.43	1.03–1.99	1.01	0.61–1.68
Partner's feeding preference				
Prefers breastfeeding	1.78	1.25–2.52	1.57	0.86–2.87
Prefers formula feeding or ambivalent	1.00		1.00	
Child characteristics				
Age received complementary foods				
Before 17 weeks	0.44	0.24–0.79	0.62	0.25–1.54
Between 17 and 25 weeks	0.65	0.39–1.08	0.77	0.39–1.55
At 26 weeks or later	1.00		1.00	
Age received formula				
Before 4 weeks	0.04	0.03–0.07	0.21	0.12–0.39
Between 4 and 7 weeks	0.03	0.01–0.05	0.08	0.02–0.35
Between 8 and 15 weeks	0.05	0.02–0.09	0.14	0.05–0.42
Between 16 and 25 weeks	0.05	0.03–0.10	0.38	0.17–0.85
At 26 weeks or later	0.15	0.08–0.28	0.20	0.07–0.53
Never received formula	1.00		1.00	

[a] Commenced but did not complete University. AOR adjusted odds ratio, BMI= Body Mass Index.

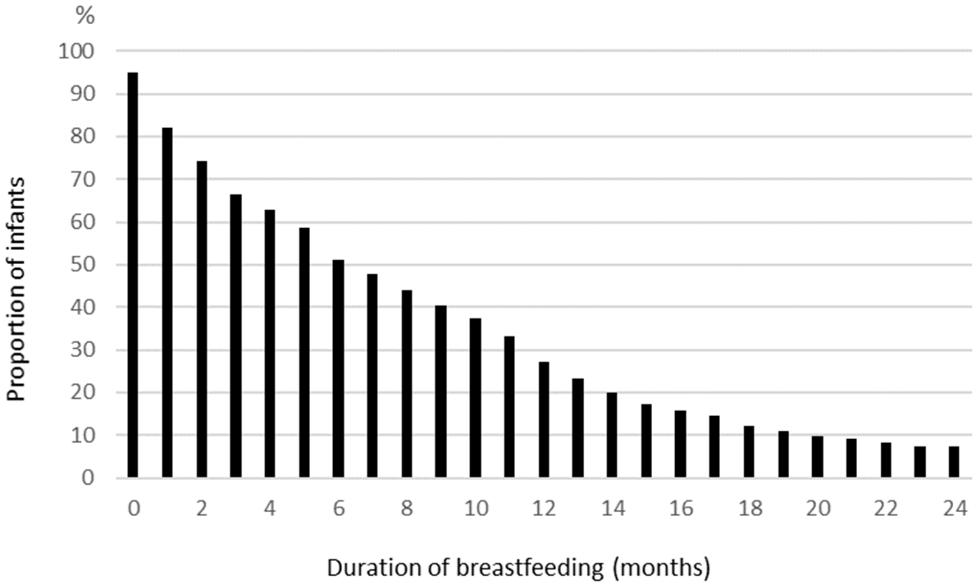

Figure S1. Proportion of children breastfed to each month of age.
